# Effects of L-type Calcium Channel Antagonists Verapamil and Diltiazem on fKv1.4ΔN Currents in *Xenopus* oocytes 

**Published:** 2013

**Authors:** Hui Chen, Dong Zhang, Jiang Hua Ren, Sheng Ping Chao

**Affiliations:** a *Department of Cardiology, Pu Ai Hospital of Wuhan City, Wuhan 430034, China. *; b *Department of Cardiology, Zhongnan Hospital of Wuhan University, Wuhan 430071, China. *

**Keywords:** Kv1.4ΔN, Potassium channels, Activation, Inactivation, Verapamil, Diltiazem

## Abstract

The goal of this study was to determine the effects of the L-type calcium channel blockers verapamil and diltiazem on the currents of voltage-gated potassium channel (fKv1.4ΔN), an N-terminal-deleted mutant of the ferret Kv1.4 potassium channel. Measurements were made using a two electrode voltage clamp technique with channels expressed stably in Xenopus oocytes. The fKv1.4ΔN currents displayed slow inactivation, with a half-inactivation potential of –38.38 mV and slow recovery from inactivation (τ = 1.90 seconds at –90 mV). The fKv1.4ΔN currents exhibited state-dependent blockade by both drugs, and the inhibition was frequency-, voltage-, and concentration-dependent, consistent with open channel block. Verapamil and diltiazem blocked fKv1.4ΔN currents with 50% inhibitory concentration (IC_50_) values of 260.71 ± 18.50 μmol/L and 241.04 ± 23.06 μmol/L, respectively. Verapamil accelerated the C-type inactivation rate and slowed recovery of the fKv1.4Δ N channel, while shifting the steady activation curve to the right. Blockade of fKv1.4ΔN currents by diltiazem was similar to that of verapamil, but diltiazem accelerated the decay rate of inactivation of fKv1.4ΔN currents without modifying the kinetics of current activation. The present results suggest that verapamil and diltiazem accelerate the C-type inactivation and slow the recovery of the fKv1.4ΔN channel in the open state.

## Introduction

The L-type calcium channel antagonists verapamil and diltiazem have been used widely for the treatment of hypertension, cardiac arrhythmia, and coronary vasospasm, and are effective in the treatment of angina pectoris (1, 2). In addition to L-type calcium channels, several voltage-gated potassium (Kv) channels are blocked by these antagonists. Verapamil has been reported to produce potent use- and frequency-dependent block of hERG channels, (3) and to inhibit the hKv1.5 channel in low micromolar concentrations (4). Diltiazem may inhibit hKv1.5 and Kv4.3 currents by binding to the open and inactivated states (5-7). Verapamil has recently been shown to inhibit Kv1.4 currents expressed in *Xenopus oocytes *(6, 8). We also found that diltiazem may block the Kv1.4 channel currents, (9) but the detailed characteristics of this effect have not been studied. 

Kv1.4 channels are expressed in the subendocardium of human and ferret heart and in the rat ventricular septum (10-12). This channel conducts transient outward potassium currents (Ito) that contribute to the early repolarization phase of the cardiac action potential. Kv1.4 expression in the mammalian endocardium is upregulated during hypertrophy and heart failure, (13-16) and this channel plays an important role in repolarization of cardiac myocytes. Kv1.4 inactivation is controlled by N- and C-type inactivation (17) *N*-type inactivation is faster, and C-type inactivation is slower, which results from conformational changes on the extracellular side of the pore (18, 19). Recovery from inactivation is governed by the slower C-type mechanism (20). Therefore, it is important to examine the effects of drugs on C-type inactivation. 

In the present study, we studied the inhibitory effects verapamil and diltiazem on Kv1.4ΔN, an N-terminal deletion construct of Kv1.4 that lacks rapid N-type inactivation but exhibits robust C-type inactivation, (21) in order to provide a more detailed understanding of their mechanisms of action. 

## Experimental

*Molecular biology*

fKv1.4ΔN cDNA (GenBank accession no.U06156) was a gift from professor Randall L. Rasmusson (University at Buffalo, SUNY). The constructs and sequences have been described previously (20-22). Removal of residues 2-146 from the N-terminal domain of Kv1.4 results in the loss of the fast N-type inactivation but leaves slow C-type inactivation intact (20-22). Transcribed fKv1.4ΔN cRNA was prepared in vitro using an mMessage mMachine kit (T3 kit, Ambion, USA).

*Isolation of oocytes and incubation*

Mature female *Xenopus laevis *frogs were provided by the Chinese Academy of Science, Beijing, China. The frogs were cared for using standards approved by the Institutional Animal Care and Use Committee of the Wuhan University of China. Mature females frogs were anesthetized by immersion in tricaine solution (1.5 g/L, Sigma) for 30 min, and oocytes were removed surgically through a lateral incision in the lower abdomen, as described previously (9). Oocytes were digested by placing the ovarian lobes in a collagenase-containing, Ca^2+^-free oocyte Ringer’s (OR_2_) solution (mmol/L): 82.5 NaCl, 2 KCl, 1 MgCl_2_, and 5 HEPES at pH 7.4, with 1 to 1.5 mg/mL collagenase (Type I, Sigma, USA). The oocytes were then shaken gently for about 1 h and washed several times with Ca^2+-^free OR_2_ solution as described previously (23). Finally, defolliculated (stage IV) oocytes were selected and placed in ND96 solution (mmol/L): 96 NaCl, 2 KCl, 1 MgCl_2_, 1.8 CaCl_2_, and 5 HEPES at pH 7.4. Each oocyte was injected with about 25 to 50 nL fKv1.4ΔN cRNA using a microinjector (WPI, Sarasota) and incubated at 18°C in ND96 solution with 100 IU/mL penicillin for a min of 16 h.

*Electrophysiology*

The two-electrode voltage clamp technique was used for electrophysiological recordings. Microelectrodes were pulled with a two-stage puller (Narishige, Japan) and had tip resistances of 0.5 to 1.0 MΩ when filled with 3 mol/L KCl. The currents in voltage-clamp mode were recorded at room temperature using a preamplifier CA-1B (Dagan, USA) and were filtered at 2.5 KHz. Recordings were made in 2 mmol/L K^+^_o_. Verapamil and diltiazem were dissolved in distilled water to give stock solutions of 100 mmol/L. All drugs were purchased from Sigma Chemical Co. (St Louis, MO, U.S.A). Drugs were perfused for 10 min before testing in order to allow equilibration with the oocytes. After this wash-on period, a series of 500-ms depolarizing pulses (from –90 to +50 mV, 1 min) were given to ensure steady-state block before beginning the experimental protocols (22).

*Data analysis*

Data were digitized and analyzed by using pCLAMP 9.0 (Axon, USA). Further analysis was performed using Clampfit 9.0 (Axon, USA) in combination with Microsoft Excel (Microsoft, USA) and Origin 6.0 software (Microcal Software, USA). Concentration-response curves were generated using the Hill equation. Inactivation time constants were obtained using a single or double exponential decay model fitted to the raw current tracings. Steady-state activation and inactivation were fitted to the Boltzmann equation, and the time course of recovery from inactivation was fitted to a single exponential equation. A paired Student’s t-test was used for statistical analysis of the data. The results are expressed as mean ± SEM, and values of p < 0.05 were considered significant. 

## Results

*Inhibition of the fKv1.4ΔN currents by verapamil and diltiazem*

**Figure 1:**
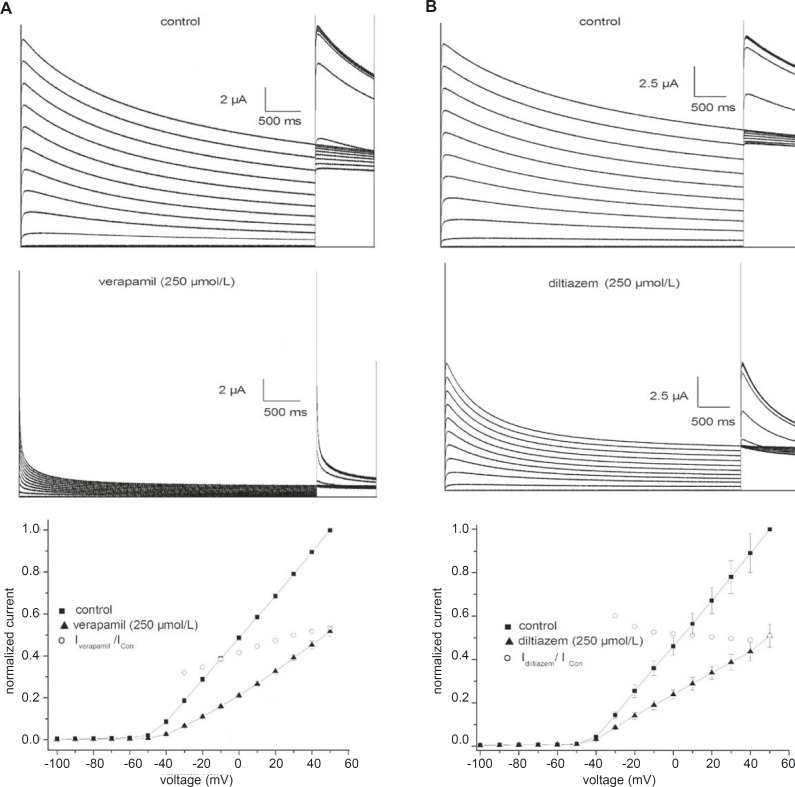
Effects of verapamil (A) and diltiazem (B) on fKv1.4ΔN channels expressed in Xenopus oocytes. Representative curves are shown for 5-second depolarizing pulses from -90 mV to voltages between –100 and +50 mV in steps of 10 mV. Upper panels: traces recorded under control conditions. Middle panels: current traces obtained in the presence of 250 μmol/L verapamil or 250 μmol/L diltiazem. The peak currents in the presence of verapamil and diltiazem were poorly resolved from the capacitance transient on this time scale. Bottom panels: effects of 250 μmol/L verapamil and 250 μmol/L diltiazem on the peak current-voltage (I-V) relationships. Currents were normalized to the peak current at +50 mV under control conditions. The IDrug/IControl ratio was plotted as a function of the membrane potential. Data are shown as mean ± SEM (n = 5).

Figure 1 shows traces of currents from fKv1.4ΔN channels expressed in Xenopus oocytes under control conditions and in the presence of verapamil (A) or diltiazem (B). The cells were held at –90 mV and given 5-sec depolarizing pulses from –100 to +50 mV. Outward currents were followed by outward tail currents upon repolarization to +50 mV. Under control conditions (Figure 1, upper panels), positive depolarization to –40 mV elicited outward currents that declined slowly during the maintained depolarization (slow C-type inactivation). The middle panels of Figure 1 show the differential effectiveness of verapamil and diltiazem at an identical concentration (250 μmol/L). Both drugs not only reduced the current amplitude, but also accelerated the inactivation. Verapamil and diltiazem reduced by 53.5 ± 6.2% and 52.2 ± 4.6% (n = 5), respectively, the peak current elicited by pulses to +50 mV. The peak currents in the presence of verapamil and diltiazem were poorly resolved from the capacitance transient on the time scale. Verapamil and diltiazem showed similar potencies in inhibiting fKv1.4ΔN currents, and the inhibitory effects were completely abrogated by a 10 min washout of drug (data not shown). 

The effects of 250 μmol/L verapamil and 250 μmol/L diltiazem on the peak current-voltage (I-V) relationships for the fKv1.4ΔN channel are shown in Figure 1 (bottom panel). The I-V relationships were constructed by plotting the normalized currents as a function of the membrane potential. Verapamil and diltiazem induced a voltage-dependent inhibition of the fKv1.4ΔN currents. To quantify the voltage dependency of fKv1.4ΔN block, the relative current (I_Drug_/I_Con_) was plotted as a function of the membrane potential. The current begins to activate at the activation threshold (between –40 and –20 mV), and both drugs decreased the peak current at potentials positive to the level of the activation threshold. The blockade remained constant at voltages above –20 mV. 

*Verapamil and diltiazem inhibit the fKv1.4ΔN currents in a concentration-dependent manner*

**Figure 2:**
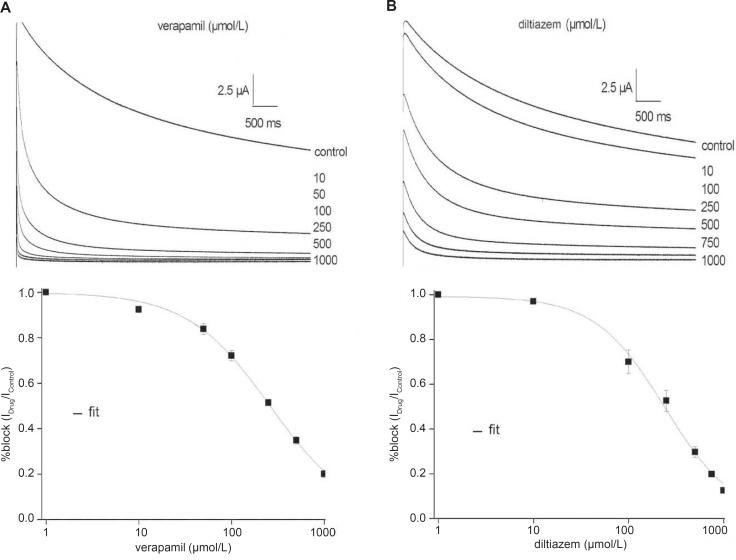
Concentration-response relationships for the inhibition of the fKv1.4ΔN currents by verapamil (A) and diltiazem (B). Upper panels: Representative current traces were elicited in the absence and presence of increasing concentrations of verapamil (A) and diltiazem (B). Currents were recorded by depolarizing pulses to +50 mV from a holding potential of –90 mV. Lower panels: The peak currents were normalized the maximum peak current under control conditions and plotted against verapamil (A) and diltiazem (B) concentrations. The curves were derived by fitting data to the Hill equation: f = K_D_/ (K_D_ + D), where f is fractional current, K_D_ is the apparent dissociation constant, and D is the drug concentration. Symbols and error bar are mean ± SEM (n = 5).

Figure 2 shows representative fKv1.4ΔN currents superimposed on the variable concentrations of verapamil and diltiazem, between 0 and 1000 μmol/L, in order to evaluate concentration-dependency. The fKv1.4ΔN currents were recorded by depolarizing pulses to +50 mV from a holding potential of –90 mV. For steady-state inhibition, a nonlinear least-squares fit of concentration-response data at +50 mV to the Hill yielded a K_D_ value of 260.71 ± 18.50 μmol/L (verapamil) and 241.04 ± 23.06 μmol/L (diltiazem) (n = 5). 

*Use-dependent block of the fKv1.4ΔN currents by verapamil and diltiazem*

**Figure 3:**
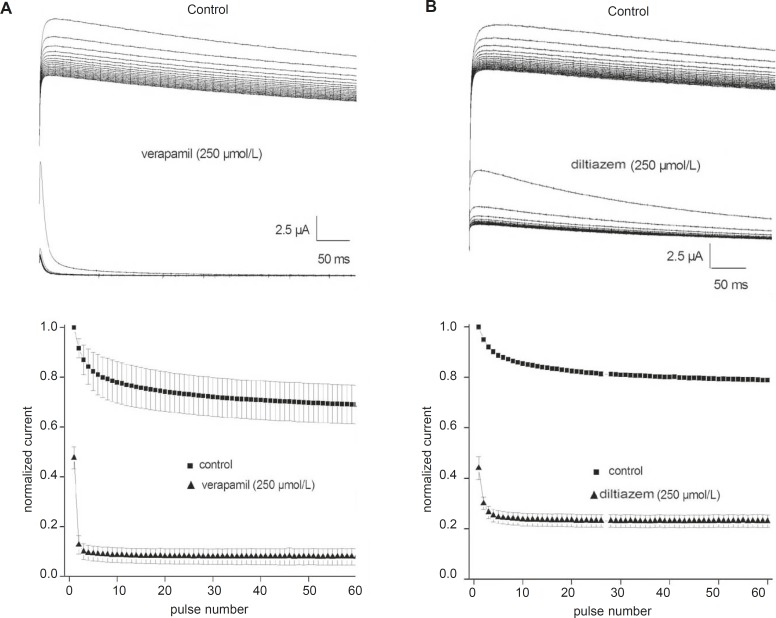
Use-dependent block of fKv1.4ΔN currents by verapamil (A) and diltiazem (B). Upper panels: Sixty repetitive depolarizing pulses from –90 to +50 mV for 500 ms each were applied in the absence and in the presence of verapamil (A) and diltiazem (B). Lower panels: Each peak current was normalized to the peak current at the first pulse under control conditions and then plotted against the number of pulses. Data are shown as mean ± SEM (n = 5).

Although 250 μmol/L verapamil and 250 μmol/L diltiazem induced approximately 50% steady-state block, this degree of block may not be attained during a single action potential. Therefore, we tested whether channel blockade by verapamil and diltiazem displayed use-dependence. Trains of 60 depolarizing pulses of 500 ms duration from –90 to +50 mV were applied at a stimulation frequency of 1 Hz, with a 1 min rest period between successive trains. The top panels in Figures 3A and B show original current records obtained after applying a pulse train protocol in the absence and presence of either 250 μmol/L verapamil or 250 μmol/L diltiazem, respectively. The peak currents were normalized to the maximum control value without drug and plotted in the bottom panels (Figure 3). Under control conditions, the fKv1.4ΔN currents were decreased by 20- 30%. However, fKv1.4ΔN currents in the presence of verapamil and diltiazem showed greater decay than under control conditions until reaching a steady-state block. The peak amplitudes of the fKv1.4ΔN currents after 60 depolarizing pulses were reduced by 83.4% in the presence of verapamil and by 47.8% in the presence of diltiazem. The degree of use-dependent block induced by verapamil was significantly greater than that induced by diltiazem. 

**Figure 4:**
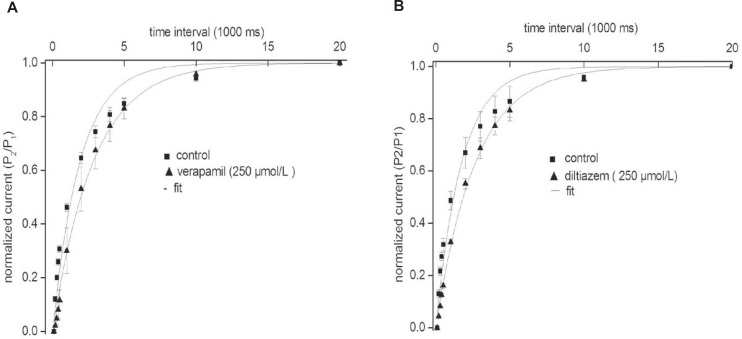
Effects of verapamil (A) and diltiazem (B) on the kinetics of the fKv1.4ΔN channel recovery from steady inactivation. The degree of recovery was measured by following a standard variable interval gapped pulse protocol. An initial 5-second pulse (P_1_) from –90 to +50 mV was followed by a second pulse (P_2_) to +50 mV after an interval of between 0.1 and 20 sec. The ratio of the peak current elicited by the P_1_ and P_2_ pulses (P_2_/P_1_) is plotted as a function of the various interpulse intervals. The continuous line represents the fit of the data to the equation: f = 1 - A*exp(–τ/t), where t is duration (in sec), τ is the time constant, A is the amplitude of the current. Data were normalized between 0 and 1 presented with intervals on a log scale. Data are shown as mean ± SEM (n = 5).

Our results indicate that the recovery of the fKv1.4ΔN channel in the presence of either verapamil or diltiazem was slower than that in the absence of drugs. To investigate this result in more detail, we measured the recovery time from inactivation using a standard gapped-pulse protocol with a variable inter-stimulus interval. The ratio of the magnitude of the first to the second pulse peak current was used as an indication of the degree of the recovery from inactivation. Figure 4 shows representative curves that were fitted with a mono-exponential function in the absence and in the presence of either 250 μmol/L verapamil (A) or 250 μmol/L diltiazem (B). The recovery time constant was slower in the presence of both drugs: (verapamil) 2.84 ± 0.17 sec, (control) 1.92 ± 0.12 sec (n = 5, p < 0.05); and (diltiazem) 2.66 ± 0.14 sec, (control) 1.73 ± 0.10 sec (n = 5, p < 0.05). Our findings showed that the rates of recovery of the fKv1.4ΔN channel from inhibition by verapamil and diltiazem were slower than the transition rate between the open and closed states in the absence of drugs, which may indicate that both drugs evoke use-dependent inhibition of fKv1.4ΔN currents. 

*Effects of verapamil and diltiazem on the activation of the fKv1.4ΔN currents*

**Figure 5:**
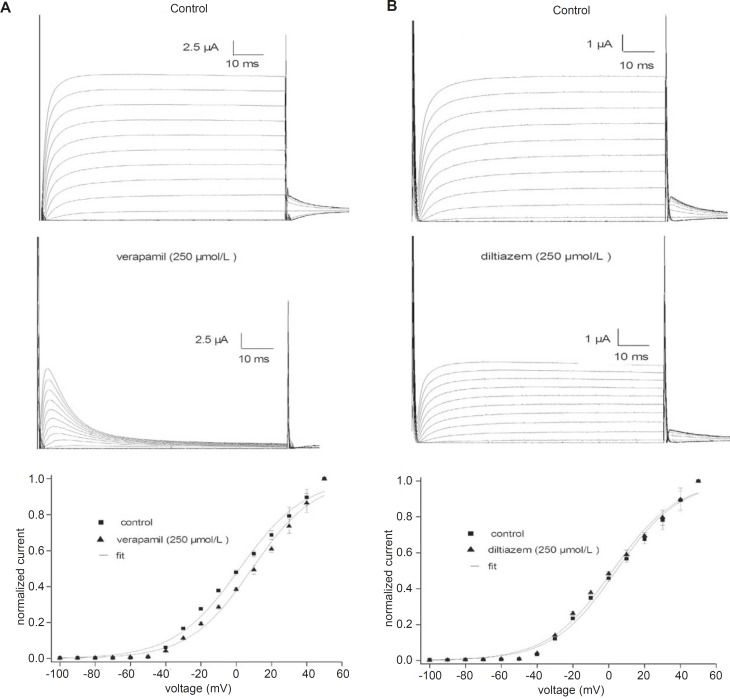
Effects of verapamil (A) and diltiazem (B) on the activation of the fKv1.4ΔN currents. Current traces were obtained by applying 80 ms pulses to potentials ranging from –100 to +50 mV and were followed by tail currents upon repolarization to –40 mV in the absence (upper panels) and in the presence of 250 μmol/L verapamil and 250 μmol/L diltiazem (middle panels). Bottom panels: steady-state activation relationships. The peak currents (measured at +50 mV) are plotted as a function of the holding potential. Continuous lines represent the fit of the data to a Boltzmann equation: f = 1/{1 + exp*[(V – V_1/2_)/k)]}, where V represents the test potential, V_1/2_ is the mid-point of activation, and k is the slope factor. Average data are shown as mean ± SEM (n = 5).

Figure 5 shows representative currents and steady-state activation relationships. The activation curves were measured in the absence and presence of either 250 μmol/L verapamil (A) or 250 μmol/L diltiazem (B), respectively. The peak currents were normalized to the peak current measured at +50 mV and were plotted as a function of the holding potential. 

We found that verapamil shifted the fKv1.4ΔN steady-state activation curve to the right, with a significant change in V_1/2_: V_1/2 Control_ = 1.94 ± 1.10 mV; V_1/2 Verapamil_ = 8.97 ± 1.08 mV (n = 5). However, diltiazem did not shift fKv1.4ΔN steady activation curve, and had no significant effect on V_1/2_: V_1/2 Control _= 4.03 ± 1.04 mV; V_1/2 Diltiazem_ = 2.08 ± 1.05 mV (n = 5). Thus, there was no effect of diltiazem on the steady activation of the fKv1.4ΔN channel. 

*Changes in the inactivation kinetics of the fKv1.4ΔN currents by verapamil and diltiazem*

To evaluate whether verapamil affects the kinetic properties of the fKv1.4ΔN currents, we analyzed the time course of inactivation and the steady-state inactivation curve in the absence and presence of 250 μmol/L verapamil (Figure 1). After exposure to 250 μmol/L verapamil, inactivation of the fKv1.4ΔN channel was clearly accelerated. Under control conditions, the inactivation of fKv1.4ΔN fitted well to a single exponential function with a time constant of 2.20 ± 0.07 seconds at +50 mV (n = 5). After exposure to 250 μmol/L verapamil, the time constant was 0.27 ± 0.04 seconds at +50 mV (n = 5). Diltiazem also produced the same effect, as described previously in detail by us (9). After addition of 250 μmol/L diltiazem, the time constant was 1.78 ± 0.29 seconds at +50 mV (n = 5). 

**Figure 6:**
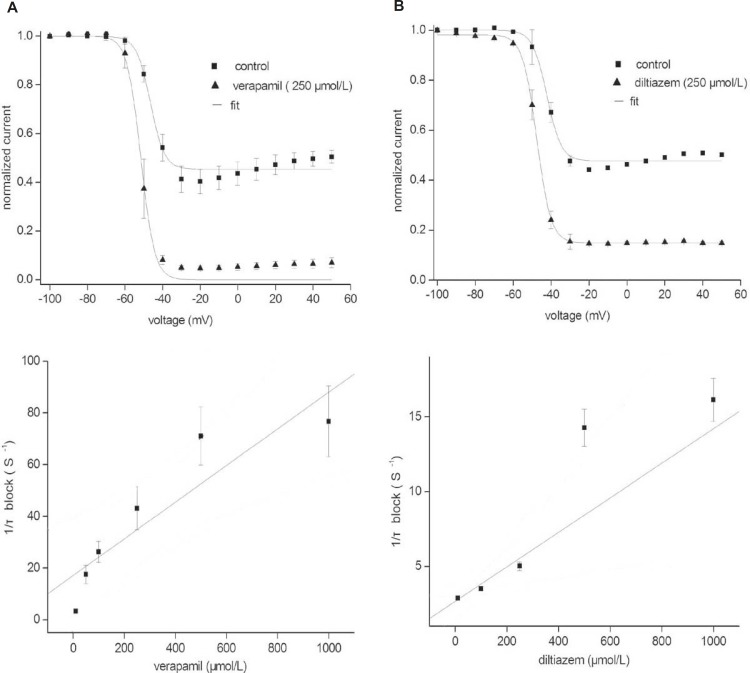
Changes in the inactivation kinetics of fKv1.4ΔN currents by verapamil (A) and diltiazem (B). Upper panels: Steady-state inactivation was studied using a two-pulse voltage protocol. Currents were measured at +50 mV, and the 5-sec pre-pulses to potential varied from –100 to +50 mV in steps of 10 mV. The curves for steady-state inactivation were fitted with the Boltzmann equation: f = 1/{1 + exp*[(V – V_1/2_)/k)]}. Where V represents the test potential, V_1/2_ is the mid-point of inactivation, and k is the slope factor. Lower panels: time constant of inhibition as a function of the drug concentration. Time constants (τ_block_) were estimated from a single or double exponential fits to the tracings shown in Figure 2. The apparent rate constants for association (k_+1_) and dissociation (k_–1_) were obtained from the equation: 1/τ_block_ = k_+1_[d] + k_–1_. Data are shown as mean ± SEM (n = 5).

We then examined and compared the effects of verapamil and diltiazem on the steady inactivation curve for the fKv1.4ΔN channel (Figure 6). The membrane potential was held at various levels between –100 mV and +50 mV, and tail currents were obtained upon repolarization to +50 mV. The peak currents were normalized, plotted against the membrane potential, and fitted to a Boltzmann equation. The steady-state inactivation relationships were normalized, as shown in Figure 6 (upper panels). In the presence of 250 μmol/L verapamil, the steady inactivation curve was shifted to the left: V_1/2 Control_ = -41.47 ± 4.25 mV; V_1/2 Verapamil _= -50.83 ± 4.58 mV (n = 5, p < 0.05). Although 250 μmol/L diltiazem also slightly shifted the steady inactivation curve to the left, V_1/2_ was not obviously changed: V_1/2 Control_ = -38.38 ± 0.81 mV; V_1/2 Diltiazem _= –39.23 ± 0.85 mV (n = 5, p > 0.05). These results indicate that verapamil but not diltiazem alters the voltage-dependency of the steady-state inactivation of the fKv1.4ΔN channel.

Inactivation of the fKv1.4ΔN currents was best fitted by a single exponential function with a time constant of 2.20 ± 0.07 seconds at +50 mV (n = 5). In the presence of verapamil, the inactivation was best fitted to a bi-exponential function, as the inactivation processes were composed of fast and slow components. The slow component (τ_slow_) was regarded as intrinsic C-type inactivation of the fKv1.4ΔN channel. The fast component (τ_fast_) was thought to represent the time constant for drug-induced blockade of the fKv1.4ΔN currents. After application of 250 μmol/L verapamil, τ_fast_ was 0.01 ± 0.002 sec and τ_slow_ was 0.27 ± 0.04 seconds (at +50 mV, n = 5). In the presence of diltiazem, the inactivation of fKv1.4ΔN was well fitted to a bi-exponential function, with τ_fast_ = 0.41 ± 0.04 sec and τ_slow_ = 1.78 ± 0.29 seconds at +50 mV (n = 5). We found that 250 μmol/L verapamil and 250 μmol/L diltiazem accelerated intrinsic C-type inactivation. 

The bottom panels of Figure 6 show τ_fast_ at +50 mV plotted against the various drug concentrations (τ_fast_ values were not shown). From this fit (left panel), in the presence of verapamil, an apparent association (k_+1_) of 0.07 ± 0.02 μmol/L^–1^s^–1^, and (k_–1_) of 17.16 ± 7.67 s^–1^ were obtained. The k_D _value derived on the basis of a first-order reaction between the drug and the channel was 242 μmol/L in the presence of verapamil. The k_D_ value of diltiazem was 267 μmol/L, as described in our previous studies (9).

## Discussion

L-type calcium channel antagonists, represented in this study by the phenylalkylamine verapamil and the benzothiazepine diltiazem, have distinct effects on cardiac electrophysiology and arrhythmias (24). They promote both use- and voltage-dependent blockade of L-type calcium channels, and a wide range of results have been reported on the concentration dependence of the blockade (3, 25). In general, phenylalkylamines block native and cloned L-type calcium channel with IC_50_ values ranging from 250 nmol/L to 15.5 μmol/L or higher. Benzothiazepines block L-type channels over a similar concentration range. 

The effects of calcium channel antagonists on other channels have also been reported. For example, verapamil inhibits rapidly activating delayed rectifier potassium channels expressed in human embryonic kidney cells with an IC_50_ of 45 μmol/L (4).Verapamil also produces high-affinity blockade of hERG currents, with an IC_50_ of 143.0 nmol/L, close to its IC_50_ for blockade of L-type calcium channels. The blockade of the hERG channel by verapamil was found to be use- and frequency-dependent(3). Verapamil has been shown to induce concentration-dependent block of currents at Kv1.1, Kv1.5, IKs, and hERG channels expressed in Xenopus oocytes with IC_50_ values of 14.0, 5.1, 161.0, and 3.8 μmol/L, respectively (26). In the human atrium, verapamil (3.2 μmol/L) was reported to reversibly suppress IK_ur_ by 50% in a concentration-dependent manner (27). However, blockade of these channels has generally required relatively high drug concentrations.

Limited data are available for the Kv1.4 potassium channel. In Xenopus oocytes, some calcium channel antagonists, including verapamil and diltiazem, have been reported to reduce fKv1.4 potassium channel currents. Our recent study showed that diltiazem inhibited the fKv1.4 potassium channel in Xenopus oocytes, (9) acting as an open channel antagonist with an IC_50_ of 241.04 μmol/L. Verapamil was also shown to reduce fKv1.4 potassium currents, (6, 8) but the drugs mechanism of action was not examined. The present observations provide novel information that verapamil inhibits fKv1.4ΔN potassium currents. Our findings agree with these reports, although the IC_50_ for verapamil block (260.71 μmol/L) is relatively higher than that for blockade of the calcium channel. However, the blockade of channels expressed in Xenopus oocytes by lipophilic drugs often requires much higher concentrations (fivefold to tenfold) than are needed to block channels expressed in mammalian cells (6). Therefore, verapamil may have a greater potency at the fKv1.4ΔN channel than reported in this study.

Our findings show that verapamil substantially inhibits currents at fKv1.4ΔN channels expressed in Xenopus oocytes in a concentration-, voltage-, and use-dependent manner. The blockade significantly accelerates channel inactivation, suggesting an open channel block (9). Diltiazem shares its characteristics. Our results demonstrate that verapamil and diltiazem exhibit the same affinity for the fKv1.4ΔN channel, but the drugs do not share high-affinity hERG channel blocking properties (3).

Since verapamil and diltiazem are L-type calcium antagonists, the electrophysiological effects of both drugs on the fKv1.4ΔN channel inactivation were determined. We discovered some differences after the application of verapamil and diltiazem. The inactivation of fKv1.4ΔN currents became biexponential, with the inactivation processes exhibiting fast and slow components. The slow component was regarded as intrinsic C-type inactivation of the fKv1.4ΔN channel, and the fast component was thought to represent time constant for drug-induced blockade of the fKv1.4ΔN currents. We found that although both drugs elicited extremely fast drug-induced inactivation representing the interaction with the open state, verapamil had a more pronounced effect on C-type inactivation than diltiazem and shifted the steady inactivation curve to the left. This may be related to a mechanism in which binding of the drug to the intracellular site of the channel triggers a conformational change at the external mouth of the pore that facilitates C-type inactivation (9, 22). Diltiazem also slightly shifted the steady-state inactivation curve to the left, but the V_1/2_ was not obviously changed. These results indicate the voltage-dependency of the steady-state inactivation of the fKv1.4ΔN channel is altered by verapamil, but not by diltiazem. The lack of effect of diltiazem on the voltage dependency of steady-state inactivation suggests that diltiazem is unlikely to interact with the inactivated state of the fKv1.4ΔN channel (28). In addition, diltiazem did not alter the steady-state activation curve, suggesting that this agent interacts with the fKv1.4ΔN channel in the open state, and not the inactivated state (29). Verapamil not only changed the voltage-dependency of the steady-state inactivation of the fKv1.4ΔN channel, but also shifted the fKv1.4ΔN steady-state activation curve to the right with a significant change in V_1/2_. Therefore, it is presumed that verapamil may interact simultaneously with the open and inactivated states of the fKv1.4ΔN channel (29). We suggest that the blockade of fKv1.4ΔN channels by verapamil is a more complex process than blockade by diltiazem. The binding of diltiazem to the channel affects C-type inactivation, however an interaction between verapamil binding and channel activation may exist (30).

Using the fast time constants in the range 10 to 1000 μmol/L, which represent the interaction of the drug with the open state, the constants k_+1_ and k_–1_ were obtained, The k_D_ values derived on the basis of a first-order reaction between the drug and channel were 242 μmol/L in the presence of verapamil and 267 μmol/L in the presence of diltiazem, respectively (9, 28, 31, 32). The values were very close to the IC_50 _values obtained from the concentration-response curve. The similarity of the IC_50_ values obtained by the two independent methods supports the open-channel block model used to calculate the rate constant for the fKv1.4ΔN channel (33, 34). 

Verapamil exerts effects on cardiac arrhythmias that can be distinguished from those of other calcium channel antagonist drugs. Although the antiarrhythmic efficacy of verapamil has usually been ascribed to its suppression of L-type calcium channels, our findings suggest that block of the fKv1.4ΔN channel also may contribute to the cardiac effects of verapamil. As mentioned in introduction, the Kv1.4 channel, which conducts Ito, contributes to phase 1 and the early part of phase 2 of the cardiac action potential, playing an important role in the repolarization of cardiac myocytes (13, 16). Suppression of the fKv1.4ΔN channel prolongs action potential duration and increases the refractory period, and may thereby terminate reentrant circuits (35). Diltiazem and verapamil share an ability to increase C-type inactivation. Blockade of fKv1.4ΔN currents and decreasing the recovery rate could attenuate the shortening of the action potential duration caused by the inhibition of L-type calcium channels by verapamil and diltiazem (9).

In the present study, we found that verapamil and diltiazem directly inhibit the fKv1.4ΔN potassium channel expressed in Xenopus oocyte by binding to the open state of the channel. Furthermore, the inhibitory effects of verapamil and diltiazem are completely independent of calcium channel inhibition. Therefore, caution should be exercised when these drugs are applied in combination with the other potassium channels blockers.
